# Complexity of Gene Expression Evolution after Duplication: Protein Dosage Rebalancing

**DOI:** 10.1155/2014/516508

**Published:** 2014-08-17

**Authors:** Igor B. Rogozin

**Affiliations:** National Center for Biotechnology Information, National Library of Medicine, National Institutes of Health, Bethesda, MD 20894, USA

## Abstract

Ongoing debates about functional importance of gene duplications have been recently intensified by a heated discussion of the “ortholog conjecture” (OC). Under the OC, which is central to functional annotation of genomes, orthologous genes are functionally more similar than paralogous genes at the same level of sequence divergence. However, a recent study challenged the OC by reporting a greater functional similarity, in terms of gene ontology (GO) annotations and expression profiles, among within-species paralogs compared to orthologs. These findings were taken to indicate that functional similarity of homologous genes is primarily determined by the cellular context of the genes, rather than evolutionary history. Subsequent studies suggested that the OC appears to be generally valid when applied to mammalian evolution but the complete picture of evolution of gene expression also has to incorporate lineage-specific aspects of paralogy. The observed complexity of gene expression evolution after duplication can be explained through selection for gene dosage effect combined with the duplication-degeneration-complementation model. This paper discusses expression divergence of recent duplications occurring before functional divergence of proteins encoded by duplicate genes.

## 1. Models of Gene Duplications

With the increasing availability of genomic data, it became clear that numerous gene families have diverged in function through series of duplications, including many lineage-specific expansions (or gene copy-number variations (CNVs) at the population level) identified in each of the genomes sequenced [1–8]. This is not surprising taking into account that gene duplications are traditionally considered to be a major evolutionary source of new protein functions [1, 2, 6, 9]. The classic concept of the functional consequences of gene duplication, proposed by Susumu Ohno, holds that duplication produces two functionally redundant, paralogous genes and, thereby, frees one of them from selective constraints. This unconstrained paralog is then free to accumulate mutations that would have been deleterious in a unique gene but become neutral after the duplication [9]. Although the most likely outcome of such neutral evolution is for one of the paralogs to fix a null mutation and become a pseudogene, there is also the possibility of fixation of mutations that lead to a new function [10–15]. One of the predictions of this neofunctionalization model of evolution of duplicate genes is the rapid loss of paralogs due to null mutations [10, 14, 15]. However, this prediction was not supported by studies on isozyme spectra of polyploids in a number of organisms [16]. Furthermore, analysis of duplicate genes in the tetraploid frog* Xenopus laevis* has shown that both copies were subject to purifying selection [17], contrary to the prediction of the neutrality of one of the copies [9]. The failure of empirical research to support Ohno's model has led to the proposal of alternative hypotheses, in particular, the general subfunctionalization model [2, 11], the more specific duplication-degeneration-complementation (DDC) model [2], and the dosage effect model [3].

The subfunctionalization hypothesis is based on the same assumptions as the Ohno's model, namely, that newly duplicate genes are redundant in function and, accordingly, a duplication event is selectively neutral [2, 11–13, 18]. However, it was argued that, as natural selection does not “know” in advance which duplicate gene should be under selection and which remains free of selective constraint, both paralogs experience a period of relaxed selection and accelerated evolution. Measurements of the selection pressure affecting paralogs shortly after duplication appear to be compatible with this reasoning [12]. During this period, both genes are likely to accumulate mutations that impair different functions of the ancestral gene, so that, after a certain point, none of the paralogs is capable of substituting for the ancestor [2, 12].

The duplication-degeneration-complementation (DDC) hypothesis is a special case of the subfunctionalization scenario. This hypothesis requires multiple losses of gene expression across tissues/cell types [2]. Under the DDC hypothesis, DNA regulatory elements are duplicated during gene duplication events. Subsequently, mutations increase specialization of gene function by degenerating modular regulatory elements in a complementary fashion in the duplicate genes, a process that is thought to contribute to the long-term preservation of duplicate genes [2]. The DDC model suggests that (1) degenerative mutations in regulatory elements and a divergence of expression patterns can increase rather than reduce the probability of duplicate gene preservation and (2) the usual mechanism of duplicate gene preservation is the partitioning of ancestral functions (e.g., expression profiles across tissues) rather than the evolution of new functions [2].

A major problem with the evolution of duplicate genes is that the creation of novel gene functions generally provides a long-term but not a short-term advantage for gene duplication. However, duplicate genes also appear to affect fitness immediately after duplication, providing a short-term advantage for duplication, conceivably through the gene dosage effect [3]. In the last decade, data have accumulated demonstrating that although a gene duplication does not necessarily double the dosage of the gene product, it nevertheless generally leads to the production of an increased amount of the product [19–21]. Some gene duplications (or gene copy-number variations (CNVs) at the population level) actually appear to be selected against due to the increase in gene dosage, including cases of gene duplications contributing to disease [5, 21–23]. Thus, the relative contributions of different factors to the evolution of paralogous genes after duplication remain a subject of intensive research and debate [7, 21, 24].

## 2. Ortholog Conjecture and Gene Duplications

The importance of appropriately designed studies to test various models of gene evolution between orthologs and paralogs was emphasized by Studer and Robinson-Rechavi [25]. It was suggested that functional changes between orthologs might be as common as between paralogs (the “uniform” model) and that more studies should be designed to test the impact of different models [25]. Robust identification of orthologs is of central importance for comparative and functional genomics due to a rarely stated but almost universally implied concept that recently has been denoted by ortholog conjecture (OC) [26]. The OC holds that orthologous genes perform equivalent functions in the respective organisms and, accordingly, experimentally determined functions of a gene can be transferred to its experimentally uncharacterized orthologs in other species (certainly, taking into account the biological differences between the organisms involved) [4, 26, 27]. Nehrt and coworkers argued that only rarely has it been noted that the OC is just a hypothesis although in most studies it is either assumed to be true or supported by evidence from a small number of genes. Therefore, Nehrt and coworkers decided to systematically test the OC hypothesis [26]. They used experiment-based annotations in the gene ontology (GO) database [28] and microarray gene expression data [29] to compare the functional and expression similarities of orthologs and paralogs in human and mouse [26]. They showed that at the same level of protein sequence divergence (i) orthologs are less similar than paralogs and (ii) between-species paralogs are less similar than within-species paralogs [26]. They further showed that (iii) functional and expression similarities between orthologs are independent of the protein sequence identity between the orthologs. These results are inconsistent with the OC hypothesis, prompting Nehrt and coworkers to propose that the primary determinant of the evolutionary rate of gene function and expression is a cellular context in which the genes act [26]. This “cellular context” hypothesis could explain why within-species paralogs were observed to be more similar in function and expression than between-species paralogs and orthologs [26].

Several consequent studies suggested that GO annotations should be used to test the OC hypothesis with a great caution [30, 31] or even should not be used for this purpose [32]. A general consensus is that GO annotations are compatible with the OC hypothesis [30, 32], although Altenhoff and coworkers suggested that GO annotations are better compatible with the “uniform” model [31]. In addition, Chen and Zhang [32] analyzed a large RNA-Seq [33] dataset of multiple tissues and showed that the expression similarity between orthologs is significantly higher than that between within-species paralogs, supporting the OC hypothesis and refuting the “cellular context” hypothesis for gene expression [32].

**Figure 1:**
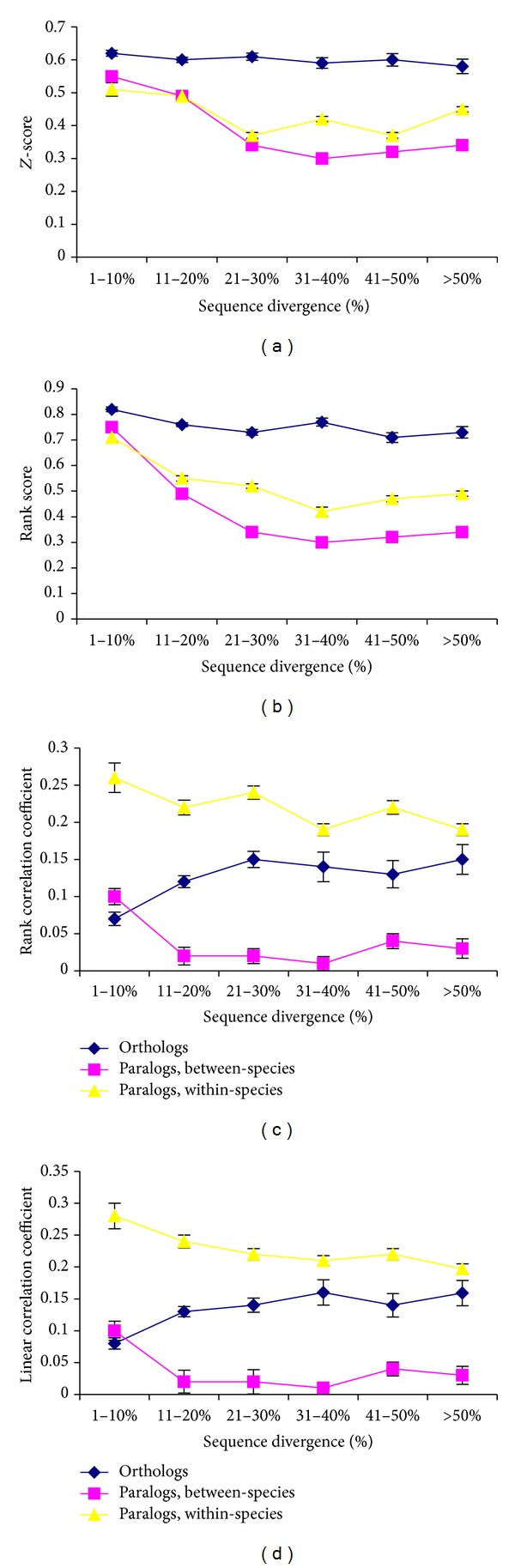
Expression and sequence similarity of orthologous and paralogous genes. (a)* Z*-score expression similarity averaged across 4 tissues. (b) Rank-based expression similarity averaged across 4 tissues. (c) Kendall's *τ* rank correlation coefficient. (d) Pearson linear correlation coefficient. The raw data is taken from Rogozin and coworkers [34]; see Table 1 for more details about procedures used in this study.

Rogozin and coworkers reanalyzed these controversial results using approaches different as much as possible from those used before and reconciled them with the literature on gene duplications [34]. This analysis of a large RNA-Seq dataset of multiple tissues from human and mouse shows that rank/*Z*-score measures of the expression similarity between orthologs are significantly higher than that between within-species paralogs (Figures 1(a) and 1(b)), supporting the OC. This result is consistent with Chen and Zhang study [32]. The plots of expression similarity measured using linear or rank correlation coefficients were qualitatively similar to the analogous plots reported by Nehrt and colleagues [26] (Figures 1(c) and 1(d)) in that the strongest correlation was observed among within-species paralogs, followed by orthologs and then by between-species paralogs. For the between-species paralogs, significant expression similarity was observed only at low sequence divergence whereas at higher divergence, the correlation coefficient values were much lower (Figures 1(c) and 1(d)). Although the correlation among within-species paralogs was high for all values of sequence divergence, it also dropped with increasing divergence (Figures 1(c) and 1(d)). It was suggested that different measures of expression similarity could reflect different salient features of gene expression, namely, tissue-specificity in the case of the correlation coefficients and relative abundance of individual mRNAs in the case of* Z*-scores and ranking scores [34]. Further analysis in which expression profiles of orthologs and paralogs were compared separately for individual gene families provided a strong argument in support of the OC [34]. However, the OC, all its importance notwithstanding, reflects only one aspect of gene evolution. The complete picture must integrate vertical descent encapsulated in the OC with the lineage-specific aspects of the evolution of paralogs; it should be interpreted also in the context of various hypotheses on evolution of gene duplications [34].

## 3. Synthetic “Protein Dosage Rebalancing” Hypothesis

The major difference between the dosage effect model and the DDC model is the role of natural selection. The dosage effect model implies that paralogs are subject to purifying selection from the onset of evolution after the gene duplication [3, 7] whereas the DDC model assumes “constructive neutral evolution” [14] whereby the paralogs are maintained due to the partial, differential degeneration of their functions resulting in functional complementarities [2, 6, 35].

**Figure 2:**
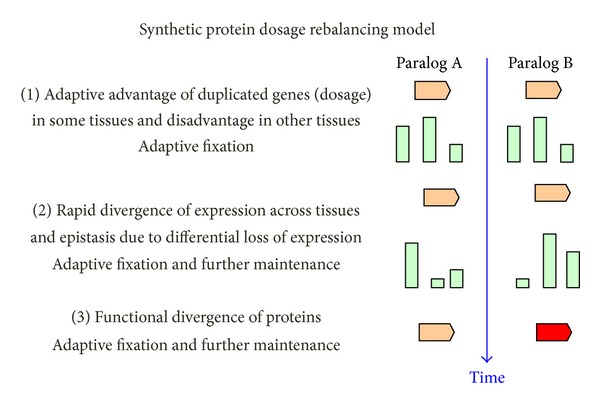
Schematic representation of the “protein dosage rebalancing” hypothesis [34]. This synthetic model is a combination of the dosage effect and DDC models: many recent gene duplications (or gene copy-number variations (CNVs) at the population level) have a positive effect in some tissues and/or environmental conditions, whereas they also have a negative effect in some other tissues and/or environmental conditions [3, 7, 21–23]. Balancing of positive and negative dosage effects influenced by natural selection may be an important factor which is causing diversification of expression patterns (rebalancing of expression) of duplicate genes in the course of fixation of gene duplications. This process is similar to the conventional dosage effect hypothesis [3]. After the gene duplication is fixed in a population, preservation of this gene duplication may be largely explained by the DDC model (maintenance of duplicate genes due to differential loss or reduction of expression in various tissues).

Results of previous studies of the “ortholog conjecture” hypothesis [26, 32, 34] are consistent with both models. (1) A significant positive correlation between gene expression and sequence divergence was found for within-species paralog measurements; this is best consistent with the dosage effect hypothesis (Figures 1(c) and 1(d)); (2) a significant difference between paralogs was found for all comparisons (Figures 1(a) and 1(b)); this is best consistent with the DDC hypothesis.

The neutrality of degenerative mutations assumed under the DDC model is amenable to a straightforward statistical test. Consider three genes, X, Y1, and Y2, in two species, among which Y1 and Y2 are lineage-specific paralogs in one species and X is the single ortholog of this pair of paralogs in the other species. Then, the neutral evolution under the strict DDC model predicts the following relationships between the expression profiles of the three genes: the profile of the gene X (*E*_x_) is expected to show a greater similarity to the combination of the profiles of the genes Y1 and Y2 (*E*_y_ = *E*_y1_ + *E*_y2_) than to either *E*_y1_ or *E*_y2_, given the differential degeneration of the expression of the two paralogs. I identified all X-Y1-Y2 triplets within the human-mouse clusters of orthologs and paralogs (see Table 1 for details) [34] and compared the expression profiles of the respective genes. The results of this analysis revealed poor agreement with the neutral prediction: in a majority of the gene triplets, *E*_x_ shows a greater similarity to *E*_y1_, *E*_y2_, or both than to the combined profile *E*_y_ although this excess is not significantly different from the uniform distribution (Table 1).

This result is consistent with many previous observations. For example, Huminiecki and Wolfe examined how the gene expression profiles of orthologous gene sets in human and mouse are affected by the presence of recent species-specific paralogs [36]. Gene expression profiles were compared across 16 homologous tissues in human and mouse genomes using microarray data for 1,575 sets of orthologous genes including 250 with species-specific duplications. It was found that there is a general trend for paralogous genes to become more specialized in their expression patterns, with decreased breadth and increased specificity of expression as gene family size increases [36]. Often, the expression of both copies of a duplicate gene is likely to have changed relative to the predicted ancestral state [36].

An interesting example of a highly redundant genome is the microcrustacean* Daphnia pulex *genome which contains at least 30,907 genes [37]. This high gene count is a consequence of an elevated rate of gene duplication resulting in tandem gene clusters. More than a third of* Daphnia*'s genes have no detectable homologs in any other available genomes, and the most amplified gene families are specific to the* Daphnia* lineage [37]. The coexpansion of gene families interacting within metabolic pathways suggested that the maintenance of duplicate genes is not random, and the analysis of gene expression under different environmental conditions revealed that numerous paralogs acquire divergent expression patterns soon after duplication events [37]. It was suggested that the persistence of some functionally divergent gene duplicates in* Daphnia* is likely to be due to preservation by entrainment (PBE) [37]. Entrainment was defined as the process of increasing the initial probability of preserving a duplicate gene through its functional interaction with existing or newly interacting genes sharing regulatory programs [37]. For example, genes with divergent expression patterns at the time of duplication, yet with regulation sufficiently similar to the expression patterns of a different interacting gene, may have combined products that are beneficial under a distinct environmental condition. In this scenario, the likelihood for preservation of these new gene duplicates is increased [37]. Thus, when genes are advantageous at the time of duplication, their coding regions are subject to purifying selection from the start and are entrained to a distinct regulatory pattern dictated by condition-specific gene-gene interactions [37]. Although the likelihood of converging on a beneficial gene expression profile near the time of duplication is small, in the case of* Daphnia*, PBE is facilitated by the high rate of gene duplication, resulting in coregulated interacting genes that can potentially define environment-specific transcriptomes, which may increase with the complexity of interactions between organisms and their environments [37].

Many studies have shown that gene duplicates in eukaryotes tend to have divergent expression patterns and that gene family expansions are associated with high levels of tissue specificity [37–44]. However, the timeframe in which these processes occur has rarely been investigated in detail, and most analyses do not include direct comparisons of orthologs as a baseline for the expected levels of tissue specificity in absence of duplications. To assess the contribution of duplications to expression divergence, Huerta-Cepas and coworkers combined phylogenetic analyses and expression data from human and mouse [42]. They analyzed differences in gene expression among human-mouse paralogs, specifically duplicated after the radiation of mammals, and compared them to pairs of orthologs in the same species. It was shown that gene duplication leads to increased levels of tissue specificity and that this tends to occur promptly after the duplication event [42].

Similar observations have been reported previously for paralogous genes in yeast [45] and fly [46]. Oakley and coworkers used a phylogenetic approach to demonstrate that the fast evolutionary rate of tissue-specific repression or loss of gene expression is significantly higher than the rate of activation or gain [46]. It was also found that DDC is consistent with only a portion of possible ancestral histories of gene expression [46]. Conceivably, the observed trend for paralogs to become more specialized in their expression patterns than expected from the strict DDC model (Table 1) as well as a significant positive correlation between gene expression and sequence divergence for within-species paralog measurements (Figures 1(c) and 1(d)) and the nonmonotonic dependency of the* Z*-scores and ranking scores on sequence divergence (Figures 1(a) and 1(b)) can be explained by selection for rebalancing of expression in different tissues and environmental conditions (Figure 2) [34]. This scenario, the “protein dosage rebalancing” [34], is consistent with several previous studies which suggest that rebalancing of expression after duplications, at least for some genes, could be beneficial [37–39, 41–44]. For example, Qian and colleagues have shown that yeast and mammalian genes often experienced a significant decrease in the level of expression after duplication. It was suggested that although the majority of the expression reduction is likely to be neutral, for some of duplicate genes, it could be beneficial through the rebalanced gene dosage [41].

## 4. Copy-Number Variations

Copy-number variations (CNVs) are alterations of a genome that results in individuals having an abnormal or, for certain genes, a normal variation in the number of copies of one or more sections of the genome. CNVs correspond to relatively large regions of the genome that have been deleted (fewer than the normal number) or duplicated (more than the normal number) on certain chromosomes. CNVs account for roughly 12% of the human genome and each variation may range from about one kilobase (1,000 nucleotides) to several megabases in size [47]. As any mutation, a duplication event by itself may have consequences on the organism's fitness. However, two factors complicate studies of the short-term immediate fitness effects of gene duplication [7]. First, the conceptual appeal of gene duplications leading to novel functions was strong enough to overshadow potential short-term fitness effects of duplications [7]. Second, there are major technical difficulties in studying CNVs that persist to this day [48, 49]. One of the most obvious problems is analysis of expression levels for recently duplicated genes and CNVs. It is not possible to use RNA-Seq reads that are mapped to two or more duplicate genes (ambiguously mapped reads); such reads are usually removed from the analysis of gene expression [32, 34]; however, this may decrease expression levels of recently duplicated genes and CNVs. This problem is even worse for the Affymetrix microarray probes that have been designed to represent the unique portions of a gene. Each probe sequence is scanned against the available genomic sequence to minimize cross hybridization between duplicate genes. This approach has a drawback of excluding many recently duplicated genes and CNVs from a microarray because unique probes cannot be designed for them [38].

CNVs were implicated in many human genetic diseases [50]; for example, it was suggested that rare CNV is an important source of risk for autism spectrum disorders (ASDs) [49, 51]. Pathogenic CNVs, often showing variable expressivity, included rare* de novo* and inherited events at over 30 gene loci, implicating several ASD-associated genes previously linked to other neurodevelopmental disorders [51]. It seems likely that the synergistic action of environmental hazards with genetic variations (including CNVs) that, in themselves, have limited or no deleterious effects but are potentiated by the environmental factors and result in dosage imbalance of neuron-specific proteins is a general principle that underlies the alarming increase in the ASD prevalence [52]. Genes affected by* de novo* CNVs converge on networks related to neuronal signaling and development, synapse function, and chromatin regulation [51]. These and many other observations of positive and negative fitness effects of CNVs [7] raised a question about validity of the so-called “backup” hypothesis (functionally redundant paralogs are used to backup important functions in the event of a severe mutation). It was suggested that the “backup” hypothesis is not supported by the analysis of expression data [41, 53]. This is consistent with the theoretical population genetic analysis by Clark [54]. It was concluded that the genetic robustness against mutations conferred by paralogous genes is a byproduct of other evolutionary processes [41]. Those processes may be extremely complicated; for example, in several cases, it appears that a gene duplication that is adaptive under a stressful condition comes at a fitness cost in a benign environment [7, 55].

## 5. Concluding Remarks

The concept of genetic balance traces back to the early days of genetics. Additions or subtractions of single chromosomes to the karyotype (aneuploidy) produced greater impacts on the phenotype than whole-genome changes (ploidy) (reviewed by [56]). Studies on changes in gene expression in aneuploid and ploidy series revealed a parallel relationship leading to the concept that many genes exhibited a stoichiometric balance, which, if upset, would modulate gene expression and protein dosage. Studies of retention of selected duplicate genes following diploidization of ancient polyploidization events have found that many duplicate genes have been preferentially maintained in a dosage-sensitive relationship [56]. Furthermore, it was hypothesized that stoichiometric alterations of macromolecular complexes or cellular networks are responsible for dominant phenotypes, because of the existing nonlinear relationships between the genotypic and phenotypic values with which they are associated [39, 57].

Many observations described in this paper are best consistent with the following possible scenario of gene duplications: many recent gene duplications (or rather gene copy-number variations (CNVs) at the population level) have a positive effect in some tissues and/or environmental conditions, whereas they also have a negative effect in some other tissues and/or environmental conditions (Figure 2) [3, 7, 21–23]. It seems likely that balancing of positive and negative dosage effects is an important factor which is causing diversification of expression patterns (rebalancing of expression) of duplicate genes in the course of fixation of gene duplications (Figure 2). This process is influenced by natural selection similar to the conventional dosage effect hypothesis [3]. After the gene duplication is fixed in a population, preservation of this gene duplication may be largely explained by the DDC model (maintenance of duplicate genes due to differential loss or reduction of expression in various tissues) that predicts that the usual mechanism of duplicate gene preservation is the partitioning of ancestral functions (expression profiles across tissues) rather than the evolution of new functions [2]. The suggested synthetic model, the “protein dosage rebalancing” model [34] (Figure 2), is a combination of the dosage effect [3] and DDC [2] models assuming importance of both natural selection and neutral evolution for maintenance of gene duplications. The “protein dosage rebalancing” model reverberates to some extent with the new mutation theory of phenotypic evolution which suggests that the driving force of phenotypic evolution is mutation, and natural selection is of secondary importance [58].

It is important to emphasize that the “ortholog conjecture,” all its importance notwithstanding, reflects only one aspect of gene evolution. The complete picture of eukaryotic evolution must integrate vertical descent encapsulated in the “ortholog conjecture” with the lineage-specific aspects of the evolution of paralogs [34, 59, 60]. This approach is embodied in a recently developed novel approach for computational annotation of gene function that incorporates information on both orthology and paralogy and yields significantly more annotations at the same average precision than a model that includes only orthologs [61].

## Figures and Tables

**Table 1:** Analysis of the duplication-degeneration-complementation (DDC) model using expression profiles of within-species paralogs (gene X versus genes Y1/Y2).

*E*_x_ − *E*_y_ > *E*_x_ − *E*_y1_, *E*_x_ − *E*_y_ > *E*_x_ − *E*_y2_ (predicted by DDC)	*E*_x_ − *E*_y_ < *E*_x_ − *E*_y1_, *E*_x_ − *E*_y_ < *E*_x_ − *E*_y2_ (contrary to DDC prediction)	*E*_x_ − *E*_y_ > *E*_x_ − *E*_y1_, *E*_x_ − *E*_y_ < *E*_x_ − *E*_y2_ Or *E*_x_ − *E*_y_ < *E*_x_ − *E*_y1_, *E*_x_ − *E*_y_ > *E*_x_ − *E*_y2_ (contrary to DDC prediction)

16	15	46

*P*_binomial_ = 0.24, for 16 (expected 0.25) versus 15 + 46 (expected 0.75)		

Kendall's *τ* rank correlation coefficient was used to measure the similarity between expression profiles of pairs of human-mouse paralogs (I analyzed cases when one genome contains one gene copy X and another genome contains two copies Y1 and Y2). The number of cases where the expression profile *E*_x_ shows a greater similarity to the combined expression profile *E*_y_ (*E*_y_ = *E*_y1_ + *E*_y2_) as predicted by the DDC model (the first column) is compared with the number of cases where *E*_x_ shows a greater similarity to *E*_y1_, *E*_y2_, or both (the second and third columns) using the binomial test. The ortholog-paralog cluster construction protocol included, first, all-against-all comparison of protein sequences from the analyzed human and genomes using the BLASTP program, with masking of low sequence complexity regions using the SEG program [34]. At the second step, orthologs were identified using symmetrical best hits. Paralogs were delineated using within-species and between-species BLASTP hits (*e*-value < 10^−20^) using the single linkage clustering procedure (the 50% identity score was used as a threshold) [34]. The RPKM values, that is, reads per kilobase of exon model per million mapped reads [33], were calculated from the counts values for each of four tissues shared by human and mouse (heart, kidney, liver, and lung) [34]. The expression data and clusters of orthologs and paralogs are available at ftp://ftp.ncbi.nlm.nih.gov/pub/managdav/paper_suppl/ortholog_conjecture/.
